# Shifting the Focus of Signaling Abnormalities in Colon Cancer

**DOI:** 10.3390/cancers14030784

**Published:** 2022-02-03

**Authors:** Markus A. Brown, Thomas Ried

**Affiliations:** Genetics Branch, Center for Cancer Research, NCI, NIH, Bethesda, MD 20892, USA; markus.brown@nih.gov

**Keywords:** WNT signaling, RAS signaling, TGF-β signaling, β-catenin, K-RAS, SMAD4, MYC

## Abstract

**Simple Summary:**

The major signaling pathways in colon cancer are WNT, RAS, and TGF-β. Components of these pathways are mutated in the majority of colon cancers, resulting in aberrantly high or low activity of the pathway. The functional consequences of the mutations reflect the behavior of these signaling pathways in intestinal stem cells. To better understand the roles of each pathway, we cover the basic function as well as points of intersection between the different pathways, to describe how they function individually, as well as together, to regulate cell proliferation.

**Abstract:**

Colon cancer tumorigenesis occurs incrementally. The process involves the acquisition of mutations which typically follow an established pattern: activation of WNT signaling, activation of RAS signaling, and inhibition of TGF-β signaling. This arrangement recapitulates, to some degree, the stem cell niche of the intestinal epithelium, which maintains WNT and EGF activity while suppressing TGF-β. The resemblance between the intestinal stem cell environment and colon cancer suggests that the concerted activity of these pathways generates and maintains a potent growth-inducing stimulus. However, each pathway has a myriad of downstream targets, making it difficult to identify which aspects of these pathways are drivers. To address this, we utilize the cell cycle, the ultimate regulator of cell proliferation, as a foundation for cross-pathway integration. We attempt to generate an overview of colon cancer signaling patterns by integrating the major colon cancer signaling pathways in the context of cell replication, specifically, the entrance from G_1_ into S-phase.

## 1. Introduction

Cancer is a disease in which cells of the body reject the commands of their tissue. This disobedience is a result of the disruption of the signaling pathways, which are no longer able to balance the activities of the cell nor effectively communicate with the extracellular environment. Signaling pathways are disrupted through a myriad of causes, though the most common is the mutation of a gene which produces a critical regulatory protein in the signaling cascade. The mutation severs the connection between the stimulus and its downstream response, because one of the proteins required to relay the appropriate signal is unable to do so. Thereby, the cell loses control over a specific aspect of its behavior and the output of the pathway becomes relatively constant, as opposed to dynamic. This influences the resting state of the cell as well as any dynamic cellular responses which require input of this (now defunct) pathway. As the mutant cell multiplies, more mutations can occur, providing the basis for selection, which results in disease progression as the cancer cell further deviates from normal cell function. Eventually, the cancer cell accumulates a sufficient number of driver mutations, approximately 5–7 in colon cancer, in various pathways, which render it ignorant of its surrounding environment. This forms the basis for metastasis, as the cancer cell is able to traverse the surrounding tissues, withstand secreted growth inhibitory signals, as well as grow in a remote site of the body, independent of the signaling pathway requirements of its tissue of origin.

### 1.1. Signaling in the Healthy Intestines

In the healthy intestinal epithelium, the WNT, EGF, Notch, and BMP pathways maintain homeostasis [1]. The intestinal stem cells are maintained by high levels of WNT, EGF, and Notch, as well as low levels of BMP signaling [2,3,4,5]. The activity of these pathways is maintained by signals from the mesenchyme surrounding the stem cell compartment, as well as other cells (such as Paneth or cKit+/Reg4+) which are intermingled with the stem cells [6,7,8]. As the stem cell progeny leave the compartment and ascend the crypt, the WNT, EGF, and Notch levels decrease, while the activity of BMP increases [9,10,11]. The changing levels of these signaling pathways results in the differentiation of the stem cells, now transit amplifying cells, into the various cell types necessary to perform the intestinal functions. Cell type is, therefore, an emergent property determined by the output of several key signaling pathways (given the context of the cell type and its epigenetic status).

### 1.2. Signaling in Colon Cancer

In colon cancer, the activities of the previously mentioned pathways are altered by mutation and closely mirror the signaling pathway arrangement found within the intestinal stem cell niche, i.e., high WNT, high EGF, and low BMP. However, Notch signaling does not appear to be heavily targeted by mutation in colon cancer [12]. Given the stochastic nature of mutation, higher mutation prevalence for specific genes is indicative of increased cellular fitness of the mutation-harboring cell compared to the surrounding normal intestinal cells, i.e., selection. Recapitulation of the stem cell state by the cancer cell indicates that the cooperativity of these pathways results in an intestinal cell with indefinite replicative potential. However, even the increased activity of a single pathway (WNT or EGF) through mutation is sufficient to result in aberrant growth and adenoma formation [13,14,15], though more faithful recapitulation of the stem cell state, i.e., increased WNT and EGF, and low BMP signaling, results in a cancer cell with malignant potential [13,16,17]. It is, therefore, the combined influence of these signaling pathways, rather than the actions of any one pathway in particular, that is necessary for the transition to malignant disease. However, cooperation between pathways does not imply synergism.

## 2. WNT Signaling

The WNT signaling pathway is an ancient mechanism necessary for guiding development and maintaining tissue homeostasis throughout multicellular life [18]. In the intestinal epithelium, canonical WNT signaling is crucial for stem cell maintenance, and thereby, homeostasis, while WNT signaling loss abrogates the intestinal proliferative compartment [2,19]. It is no coincidence that the signaling pathway most critical for maintaining the intestinal stem cell state is also the most frequently mutated in colon cancer.

### 2.1. WNT Production

WNT signaling begins with the production of a small (~40 kDa), cysteine-rich protein, referred to as a WNT [20,21]. Humans produce 19 different WNTs, which perform various developmental functions with limited functional redundancy [22,23,24,25,26,27]. As part of their maturation, WNTs are lipid-modified by the addition of palmitoleic acid, which is appended by the O-acyltransferase enzyme, Porcupine, within the endoplasmic reticulum (ER) [28,29,30,31,32,33,34]. WNT acylation is required for binding to WLS (Wntless/Evi), an essential component of the WNT secretory pathway, while inhibition of WNT acylation results in WNT accumulation within the ER [34,35,36,37,38,39,40]. Following the binding of WNT to WLS, the WNT is transported from the Golgi to the plasma membrane for secretion and transfer to its target cells via WNT-binding proteins, exosomes, or cytonemes [41,42,43,44,45]. WNT acylation renders it hydrophobic and limits diffusion through the extracellular space [30].

### 2.2. WNT Binding

**Figure 1:**
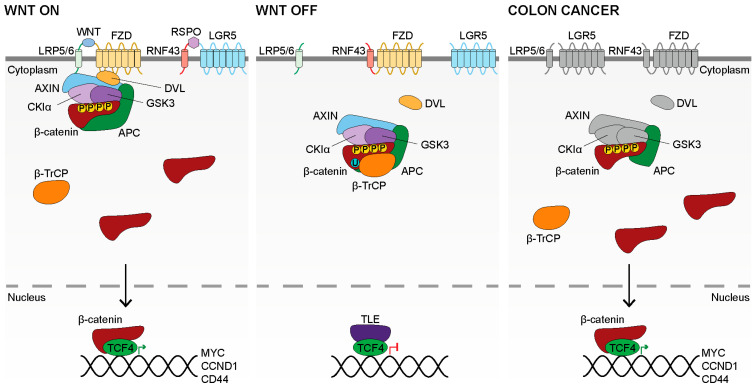
The WNT Signaling Pathway. In the **WNT ON** state (**left**), an R-spondin ligand (RSPO) binds LGR5 and RNF43/ZNRF3, preventing the inhibition of FZD. FZD is, therefore, able to bind a WNT ligand and LRP5/6 to form a co-receptor complex. DVL and AXIN, as well as the rest of the DC, is recruited to WNT–FZD–LRP at the plasma membrane. β-catenin may be phosphorylated within the sequestered DC, however, it cannot be ubiquitinated by β-TrCP, and is not degraded by the proteasome. The DCs become saturated with β-catenin, and newly synthesized β-catenin accumulates in the cytoplasm. β-catenin then migrates to the nucleus where it binds the TCF/LEF family of transcription factors (TCF4 in the intestines). The potent transcriptional activation domain of β-catenin, complexed with TCF4, results in the expression of WNT signaling target genes such as *MYC*, *CCND1*, and *CD44*. In the **WNT OFF** state (**center**), no WNT or RSPO ligands are present, and FZD is inhibited by RNF43/ZNRF3. The DC remains in the cytoplasm where it actively phosphorylates β-catenin. Phosphorylated β-catenin is recognized by β-TrCP and ubiquitinated, resulting in degradation of β-catenin (not diagrammed). β-catenin is unable to accumulate in the cytoplasm. The TCF/LEF factors are bound by the inhibitory TLE factors and repress target gene expression. In **Colon Cancer** (**right**), APC is truncated due to mutation, which compromises the ability of the DC to target β-catenin for degradation. The upstream components of the pathway are in grey as their functional role, to regulate β-catenin, has been compromised by APC truncation. β-catenin accumulates in the cytoplasm, after which it migrates to the nucleus. β-catenin binds the TCF/LEF transcription factors and constitutively activates the expression of WNT signaling target genes which leads to proliferation and the maintenance of stemness.

Once at the target cell membrane, the WNT ligand binds to a Frizzled (FZD) receptor as well as a Low-Density Lipoprotein Receptor-Related Protein (LRP) to form a FZD–LRP coreceptor, thus initiating canonical WNT signaling [46,47,48,49,50,51] (Figure 1, left). Humans have 10 FZDs, to which the 19 WNTs bind promiscuously [52,53,54]. The LRPs associated with WNT signaling are LRP5 and LRP6 [47,48,49]. Importantly, the palmitoleic acid appended to the WNT is also required for FZD and LRP binding [50,51,55]. Lipid modification is, therefore, necessary for WNT secretion, as well as receptor binding (which leads to active WNT signaling). Taken together, the hydrophobicity of the acylated WNT severely limits its diffusion in the extracellular space, enforcing strict control over which cells receive a productive WNT ligand [30,34,56]. However, additional regulatory mechanisms exist to modulate WNT–FZD–LRP binding. These include NOTUM, which functions by WNT deacylation; DKK, which functions by binding LRP and blocking WNT binding; or sFRP, which sequesters the WNT itself [55,57,58,59]. Additionally, FZD may be ubiquitinated and internalized by the transmembrane E3 ubiquitin ligases, RNF43 and ZNRF3, which block WNT signaling activity [60,61]. However, RNF43 and ZNRF3 activity can be inhibited by the intestinal stem cell marker, LGR5, in the presence of its ligand, RSPO [19,60,61,62]. RSPO and LGR5, therefore, permit WNT signal transduction by maintaining FZD at the plasma membrane for WNT binding through inhibition of RNF43 and ZNRF3.

### 2.3. WNT Signal Transduction

Upon successful WNT–FZD–LRP binding, the cytoplasmic domains of FZD and LRP become associated, which results in the recruitment of DVL and AXIN and the phosphorylation of LRP by CKIγ and GSK3, in an incompletely understood mechanism [63,64,65,66,67]. AXIN is a core component of the Destruction Complex (DC), and thus, sequestration of AXIN at FZD–LRP results in sequestration of the DC [68]. The DC is comprised of AXIN, APC, CKIα, GSK3, β-catenin, and β-TrCP [69,70,71,72,73,74]. DC sequestration inhibits β-catenin ubiquitination, but not phosphorylation, halting β-catenin turnover and saturating the DCs [68]. Since β-catenin is consistently produced, DC saturation results in β-catenin accumulation in the cytoplasm, which then migrates to the nucleus [68]. Nuclear migration of β-catenin may be facilitated by RAPGEF5 [75]. Once within the nucleus, β-catenin competes with the transcriptionally repressive TLE factors to bind with the TCF/LEF family of transcription factors, which are the canonical WNT transcription factors [76,77,78,79]. Association of the potent transcriptional activation domain of β-catenin with the TCF/LEF transcription factors results in the activation of WNT target gene expression [78,79] (Figure 1, left). The role of each TCF/LEF factor is largely distinct, though there remains limited functional redundancy, such as between TCF1 and LEF1 in the lymphoid lineage [80,81,82,83,84,85,86,87,88]. In the colon, TCF4, encoded by *TCF7L2*, is necessary for intestinal development in mouse embryos as well as intestinal maintenance in adult mice [2,83,84]. While it is difficult to comprehensively identify all crucial downstream TCF4 targets in the intestine; *MYC*, *CCND1*, and *AXIN2* are generally considered to be key transcriptional targets of an active WNT signal and serve as WNT reporter genes [89,90,91].

### 2.4. Inactive WNT Signaling

In the WNT OFF state, the FZD and LRP receptors remain unbound by WNT, and therefore, remain unassociated in the cytoplasm. FZD receptors are internalized by ZNRF3 and RNF43, blocking accessibility to WNT [60,61]. The DC remains, unrestricted, in the cytoplasm. APC facilitates β-catenin retrieval from the nucleus, as APC contains nuclear import and export domains, as well as incorporation of β-catenin into the DC via the 15- and 20-amino acid domains (β-catenin binding), and SAMP domains (AXIN binding) of APC [69,70,92,93,94,95,96]. Once within the DC, CKIα phosphorylates β-catenin at S45, which primes it for subsequent phosphorylation by GSK3 at T41, S37, and S33 [71,97]. The phosphorylated residues form a motif recognized by β-TrCP, which ubiquitinates β-catenin, targeting it for destruction by the proteasomal machinery [71,72,98]. β-catenin is rapidly degraded and does not accumulate in the cytoplasm, nor does it migrate to the nucleus. The TCF/LEF factors, therefore, remain bound by the TLE family of transcriptional repressors and inhibit WNT target gene expression [99,100] (Figure 1, center).

### 2.5. WNT Signaling in Colon Cancer

The majority (93%) of colon cancers harbor mutations in the WNT signaling pathway, which result in altered WNT signaling activity [12]. The majority of these mutations arise in *APC*, resulting in a truncated APC protein which can not efficiently facilitate the degradation of β-catenin, resulting in constitutively active WNT signaling [101,102,103,104] (Figure 1, right). *APC* appears to follow Knudsen’s two-hit theory, as both alleles have been found to be mutated in the majority of adenomas from patients with familial adenomatous polyposis (FAP) [105,106,107,108,109]. Stabilizing mutations in β-catenin also occur, rendering it refractory to phosphorylation, and therefore degradation, resulting in constitutive WNT signaling [103]. Together, *APC* and *CTNNB1* account for the majority of WNT mutations in colon cancer [12]. These mutations sever the dependence of the cell upon WNT ligands for activated WNT signaling activity, and mutant cells no longer require signals from the mesenchyme or Paneth cells to maintain active WNT. These mutations, therefore, result in the protracted maintenance of a WNT signaling phenotype outside of the intestinal stem cell niche. The *APC* and *CTNNB1* mutations are mutually exclusive, indicating that they serve equivalent roles in WNT signaling activation [110]. Unfortunately, β-catenin accesses a wide array of target genes through its association with the TCF/LEF factors, complicating our understanding of the downstream consequences of high levels of nuclear β-catenin. However, MYC appears to be the single most important target, as *MYC* deletion rescues the consequences of *APC* loss, despite high levels of β-catenin [111].

## 3. RAS Signaling

RAS signaling is crucial for mediating cellular growth, differentiation, and survival [112]. As a result of their growth and anti-apoptotic functions, members of the RAS signaling pathway, most notably *K-RAS*, are frequently mutated in cancer [113]. Approximately 20% of all cancers and 50% of all colon cancers harbor an activating mutation in *RAS* [112,113]. In colon cancer these mutations typically occur following *APC* mutation during tumorigenesis, though this order is not invariant [113].

### 3.1. RAS Signaling Receptors and Ligands

**Figure 2:**
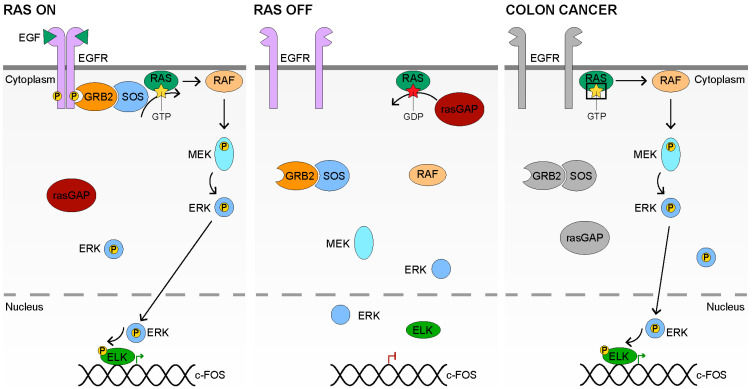
The RAS-MAPK Signaling Pathway. In the **RAS ON** state (**left**), EGF binds to EGFR with 2:2 stoichiometry at the plasma membrane. EGF binding results in dimerization of the receptors, and their autophosphorylation. GRB2 binds to the phosphorylated tyrosine on EGFR via its SH2 domain, and subsequently recruits the rasGEF, SOS. SOS catalyzes the exchange of GDP for GTP on RAS, activating it. Activated RAS then activates RAF, which phosphorylates MEK, which, in turn, phosphorylates ERK. Phosphorylated ERK travels to the nucleus, though some remains in the cytoplasm, resulting in the phosphorylation of cytoplasmic and nuclear targets. ERK phosphorylates the transcription factor ELK, which allows it to form a ternary complex with serum response factor (SRF) at serum response elements (SREs). The complex then drives the expression of growth factor response genes, such as *FOS*, a component of the AP-1 transcription factor. In the **RAS OFF** state (**center**), no EGF or EGF-like ligand is present and EGFR remains unbound, its dimerization inhibited by its conformation. GTP bound to RAS is hydrolyzed to GDP by rasGAP, inactivating it. RAS which is bound to GDP, remains so. GRB2 and SOS remain unbound to EGFR, and RAF is inactive. MEK and ERK remain unphosphorylated, with some ERK remaining in the nucleus. ELK is unphosphorylated and is unable to form a ternary complex with SRF at the SRE. In **Colon Cancer** (**right**), *K-RAS* is mutated, at codon 12, which renders it refractory to GTP–GDP exchange by rasGAP, and rasGAP is, therefore, in grey. The actions of the upstream components EGFR, GRB2, and SOS are also in grey as their purpose, promoting the GTP bound state of RAS, has been achieved. RAF–MEK–ERK is active, inducing the activity of ELK and others.

RAS signaling relies on the actions of the Epidermal Growth Factor Receptors (EGFR), which are Receptor Tyrosine Kinases (RTKs), for signal transduction through the plasma membrane. The EGFR family consists of four members: ERBB1 (EGFR), ERBB2 (HER2), ERBB3 (HER3), and ERBB4 (HER4) [114,115,116,117]. EGFR is capable of binding a variety of ligands, including Epidermal Growth Factor (EGF), Transforming Growth Factor-α (TGF-α), and heparin-binding EGF-like growth factor (HB-EGF), as well as several others [114,118,119,120,121,122]. ERBB2 has no known ligands and ERBB3 and ERBB4 bind to the neuregulins (NRG) [123,124]. The major RAS ligands in the intestinal epithelium are EGF and TGF-α [6]. EGF–EGFR binding results in EGFR dimerization into homo or heterodimers with other members of the EGFR family, such as ERBB2 [125,126,127] (Figure 2, left). The ability to form receptor heterodimers allows for a wider variety of ligand binding and diversifies the intracellular responses based on the participating receptors [128].

### 3.2. RAS Signal Transduction

Oligomerization of the EGFR family of receptors results in transphosphorylation of the dimerized, cytoplasmic receptor domains [129,130,131,132]. The phosphorylated residue Y1068 on EGFR forms a docking site for GRB2, via its SH2 domain, which recruits its binding partner, SOS, a RAS-specific Guanine nucleotide Exchange Factor (GEF) [133,134,135,136,137]. SOS activates the small guanosine triphosphatase (GTPase) RAS by inducing the exchange of GDP for GTP [138,139,140]. When activated (bound by GTP), RAS activates a variety of downstream processes involving PI3K, RALGDS, and PLCε, as well as the protein serine/threonine kinase RAF [112,141,142,143]. This review discusses the major downstream process, the MAPK cascade, which is initiated by RAF. Once activated by RAS, RAF phosphorylates, and by doing so activates, the mitogen-activated protein kinase kinase, MEK [144,145,146,147]. Subsequently, MEK phosphorylates ERK on its activation loop [146,147,148,149]. A fraction of ERK is transported to the nucleus following activation, resulting in the phosphorylation of both cytosolic and nuclear substrates [150,151,152,153]. Within the nucleus, ERK phosphorylates a variety of transcription factors, including MYC and members of the ETS family, such as ELK1 [154,155,156]. Phosphorylation of ELK1 allows it to form a ternary complex with serum response factor (SRF) at serum response elements (SREs) [155,156]. A notable transcriptional target of ELK1 is *FOS*, a member of the AP-1 transcription factor [155,156]. Additionally, SREs are present within many immediate early response genes [157]. RAS signaling therefore results in the expression of key cell-cycle regulatory proteins which promote proliferation and survival.

### 3.3. Inactive RAS Signaling

In the absence of EGF or an EGF-like ligand, EGFR does not dimerize, since the dimerization interface remains sequestered until EGF binding, which induces a conformational change in the receptor [132,158]. EGFR dimerization alters the conformation of the kinase domains which permits phosphorylation [159,160]. Following EGFR autophosphorylation, the EGF–EGFR complex is rapidly internalized and subsequently degraded, limiting the amount of RAS activation [161,162,163]. RAS proteins contain intrinsic GTPase activity and gradually exchange GTP for GDP, leading to their own inactivation, a process greatly facilitated by the GTPase-activating proteins (GAPs) [164,165,166] (Figure 2, center).

### 3.4. RAS Signaling in Colon Cancer

In colon cancer, *K-RAS* mutations, which occur almost exclusively in codons 12, 13, or 61, are found in approximately 50% of tumors [12,113]. These mutations alter the residues surrounding the GTP–GDP exchange site on RAS, inhibiting the ability of rasGAPs to hydrolyze GTP and return K-RAS to the GDP-bound state [167,168] (Figure 2, right). RAS signaling, therefore, remains persistently active in *K-RAS* mutant cells and significantly reduces the reliance of the cell upon EGF and EGFR for an active RAS growth signal. Mutations in *K-RAS* are mutually exclusive with mutations in *N-RAS* or *B-RAF*, implying similar regulatory functions [12,169]. Consistent activation of RAS signaling results in the phosphorylation of ERK targets, such as the ETS transcription factors, which are necessary for the transforming effects of RAS [155,156]. ELK1 phosphorylation leads to ternary complex formation and the expression of *FOS* [155,156]. The other major component of the AP-1 transcription factor, *JUN* is also mediated by RAS signaling; however, it relies upon the JNK cascade [170,171,172]. Tumors containing an activated RAS pathway display increased proliferation through the actions of the ETS factors and AP-1 [112].

## 4. TGF-β Signaling

TGF-β signaling influences a diverse array of cellular processes throughout the lifespan of the organism, including proliferation, differentiation, morphogenesis, and regeneration [173]. In the colonic epithelium, finely regulated gradients of TGF-β, alongside WNT, mediate differentiation of the intestinal stem cells and transit-amplifying cells [1]. In colon cancer, mutations which influence TGF-β are typically the last perturbation preceding malignant transformation [174].

### 4.1. TGF-β Ligands and Receptors

**Figure 3:**
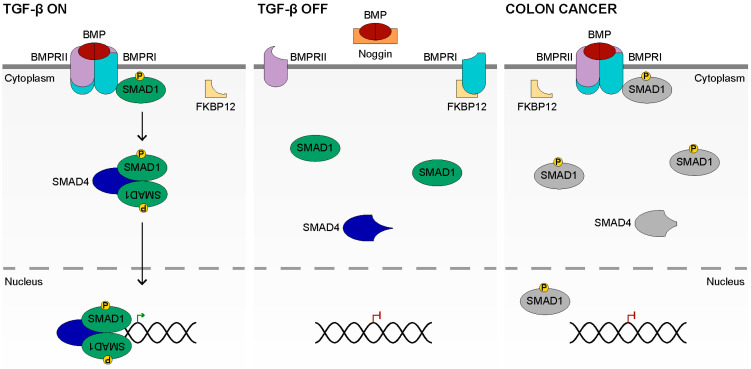
The TGF-β Signaling Pathway. In the **TGF-β ON** state (**left**), a BMP dimer binds to two BMPRI and two BMPRII to from a hetero-tetrameric complex. The Type II receptor phosphorylates the Type I receptor (not shown), which removes the inhibitory FKBP12 protein. An R-SMAD, such as SMAD1, binds the Type I receptor and is phosphorylated. The phosphorylated R-SMAD then forms a hetero-trimeric complex with SMAD4, the Co-SMAD and another SMAD1. The complex migrates to the nucleus where it binds the DNA and influences target gene expression. In the **TGF-β OFF** state (**center**), the BMP dimer is bound by Noggin which blocks binding to the Type II or Type I BMP receptors. The receptors remain unassociated, which prevents the phosphorylation of BMPRI. Therefore, BMPRI remains bound by FKBP12, which inhibits the phosphorylation of the R-SMAD. SMAD4 does not form a complex with the R-SMAD. In **Colon Cancer** (**right**), with a mutated *SMAD4*, the BMP ligand remains capable of inducing hetero-tetramer formation and phosphorylation of the R-SMAD. However, the mutation inhibits formation of the Co-SMAD::R-SMAD complex and the R-SMAD and Co-SMAD is therefore in grey. SMAD1 may migrate to the nucleus alone, however the intended function of the Co-SMAD::R-SMAD complex is not performed.

The TGF-β family of ligands constitutes the largest family of secreted morphogens (33 members) and can be divided into the TGFβ–Activin–Nodal and BMP subfamilies [175]. These ligands are produced by cleavage of a pro-domain, which releases the mature domain of the ligand [176,177]. The mature ligand dimerizes, which is essential for receptor activation [178] (Figure 3, left). These dimeric ligands bind to the serine/threonine kinase family of receptors [179,180,181]. Humans utilize seven Type I and five Type II receptors, which are dedicated to TGF-β signaling [182,183]. In the intestines, the major TGF-β ligands are BMP2 and BMP4, which bind the Type I receptors BMPRIA and BMPRIB as well as the Type II receptor, BMPRII; however, the TGF-β subfamily is active as well [184,185].

### 4.2. TGF-β Signal Transduction

The intracellular workhorses of the TGF-β signaling pathway are the SMAD proteins. There are three functional classes of SMADs: receptor-regulated (R-SMAD), co-mediator (Co-SMAD), and inhibitory (I-SMAD) [186]. The R-SMADs include SMAD1, 2, 3, 5, and 8, and the Co-SMAD is SMAD4, while the I-SMADs are SMAD6 and 7 [182,186]. SMAD2 and 3 transduce the TGF-β ligand message, while SMAD1, 5, and 8 transmit the BMP message. BMP ligand binding cooperatively brings together two Type I and Type II BMP receptors, forming a hetero-tetramer [186]. Complex formation allows the Type II receptor to phosphorylate the Type I receptor’s GS domain, facilitating the exchange of the inhibitory FKBP12 for an R-SMAD(1, 5, 8) [187,188,189,190]. R-SMAD localization to the receptor is facilitated by the protein SARA [191]. Upon R-SMAD binding, the Type I kinase domain phosphorylates the R-SMAD, after which it dissociates from the receptor [187,192,193]. Phosphorylated R-SMADs then form a hetero-trimeric complex with SMAD4, the Co-SMAD [194,195]. The SMAD complex then translocates to the nucleus, where it regulates target gene transcription alongside various nuclear cofactors [187,193,196]. These nuclear co-factors bind the R-SMAD, ensuring the specificity of the transcriptional response [182]. TGF-β target genes include p15^Ink4b^ and p21^Cip1^, the cyclin-dependent kinase inhibitors which mediate the growth-repressive actions of TGF-β [197,198,199].

### 4.3. Inactive TGF-β Signaling

TGF-β signaling is regulated at several levels. At the ligand level, Noggin and Chordin, are both capable of binding to the BMP ligands, which prevents their association with the BMP receptors [200,201,202] (Figure 3, center). When the receptors remain unassociated, either through a lack of BMP ligand or the presence of Noggin or Chordin, FKBP12 binds to, and inhibits, the phosphorylation of the Type I receptors [203]. The R-SMAD is unable to bind to the Type I receptor and the Co-SMAD::R-SMAD complex is not formed. The I-SMADs also negatively regulate TGF-β signaling by competing with R-SMADs for receptor binding or with SMAD4 complex formation [204,205]. SMAD6 inhibits BMP signaling, while SMAD7 can inhibit either TGF-β or BMP signaling.

### 4.4. TGF-β Signaling in Colon Cancer

TGF-β signaling negatively regulates cell proliferation in the colon and is often mutated in colon cancer [13,206]. These mutations, which occur in *TGFBRII*, *SMAD2*, or *SMAD4* inactivate the protein and abrogate the ability of TGF-β to regulate proliferation [206,207,208,209] (Figure 3, right). Mutations in *SMAD4* (10%) are more common than mutations in *SMAD2* (6%); however, in hypermutated tumors, mutations in *TGFBRII* were observed in 51% of tumors [12]. The *SMAD4* mutations may occur within the MH2 domain, which inhibits the ability of SMAD4 to bind the R-SMAD and transfer to the nucleus, or may occur within the MH1 domain, which results in autoinhibition of the SMAD, which also inhibits SMAD complex formation [210,211]. Ultimately, biallelic mutation of the TGF-β signaling pathway components results in decreased regulation of cell growth and is typically the last mutation to occur prior to the transition to malignancy [212].

## 5. The Cell Cycle in Cancer

The cell cycle is initiated by progression of the cells past the restriction point in G_1_ [213,214]. Once this point has been passed, no additional external stimuli are required for progression through the cell cycle [213,214]. The restriction point represents the shift from a reliance on cyclin D-CDK4/6 for progression to S-phase, to a reliance on cyclin E-CDK2 [214,215,216]. The goal of the cancer cell is, therefore, to progress through the restriction point by activating cyclin E, which depends upon cyclin D.

## 6. WNT, RAS and TGF-β

The WNT, RAS, and TGF-β signaling pathways are frequently mutated in sporadic colon cancer [12,13]. WNT signaling is necessary for intestinal homeostasis throughout life, as loss of WNT signaling activity results in the loss of the proliferative compartment and breakdown of the intestinal epithelium in neonatal and adult mice [2,84,217]. However, when WNT signaling is aberrantly activated, the intestinal crypts exhibit exacerbated proliferation [218,219,220]. A similar, yet less drastic, effect occurs when EGF signaling is inhibited in the intestines, which induces stem cell quiescence, while EGF activation results in aberrant proliferation [221,222,223]. The role of TGF-β is to negatively regulate proliferation in the intestine, striking a balance with the proliferative actions of WNT and EGF. Loss of TGF-β regulation also results in aberrant proliferation [5,11]. Taken together, disruption of the WNT–EGF–TGFβ interaction, such as by an *APC*, *K-RAS*, or *SMAD4* mutation, results in an imbalance of the signaling pathways and confers a proliferative advantage upon the mutation-harboring cell.

**Figure 4:**
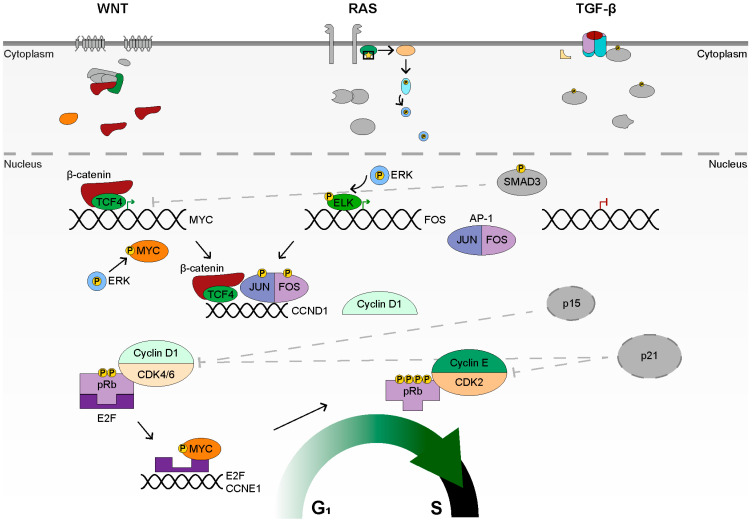
Signaling Overview in Sporadic Colon Cancer. Activated WNT signaling, via mutations in *APC* or *CTNNB1*, results in constitutive formation of βcat/TCF4 complexes, which drive WNT target gene expression, such as *MYC* and *CCND1*. Activated RAS signaling, via mutations in *K-RAS*, result in activation of ERK, which phosphorylates MYC, as well as the R-SMADs, inhibiting SMAD migration to the nucleus (not shown). ERK also phosphorylates ELK, which results in expression of *FOS*. JUN is regulated by the JNK cascade (not shown), and binds to FOS, forming the AP-1 transcription factor. AP-1 and βcat/TCF4 drive expression of *CCND1*. Cyclin D1 binds to CDK4 or CDK6 and phosphorylates pRb, causing the release of E2F. E2F, with MYC, drives the expression of other *E2F* genes and *CCNE1*. Cyclin E binds to CDK2, which fully phosphorylates pRb, leading to progression into S-phase. The TGF-β pathway is inactivated due to mutation, though low levels of phosphorylated R-SMAD may be found in the nucleus. The cell cycle inhibitors, p15 and p21, which would be expressed given active TGF-β, cannot inhibit CDK4/6 or CDK2, and are in grey.

The aberrant activation of WNT signaling leads to the constitutive formation of β-catenin/TCF4 transcriptional complexes, which enforce a crypt progenitor phenotype [103,104,224]. The downstream target genes of TCF4 include *MYC* and *CCND1*, which both facilitate progression into S-phase [89,90]. However, *MYC* appears to be the major target of WNT signaling, as *MYC* deletion rescues *APC* loss, despite high levels of β-catenin [111]. MYC lies at the core of cell cycle progression and facilitates the expression of genes associated with nucleotide biosynthesis, ribosome biogenesis, RNA processing, cell cycle progression, and DNA replication [225,226]. Additionally, MYC also represses the expression of key cell cycle inhibitors such as *CDKN1A* (p21) and *CDKN2B* (p15) [227,228]. The ability of TGF-β to counteract the WNT signal is centered on *MYC*, as *MYC* expression is repressed by SMAD3, which is necessary for TGF-β-induced G_1_ arrest [229,230]. Furthermore, TGF-β expresses *CDKN1A* and *CDKN2B*, the genes which are inhibited by MYC [197,198]. WNT and TGF-β, therefore, compete to regulate cell cycle progression at the *MYC* level, with TGF-β dominance resulting in p21 and p15 production and cell cycle arrest, while WNT dominance results in *CDKN1A* and *CDKN2B* repression. The *APC* mutation appears to give WNT a slight advantage. The increased levels of MYC, induced by activating WNT mutations, are complemented by activated RAS signaling via K-RAS [231]. Phosphorylated ELK1 leads to expression of *FOS*, a member of the AP-1 transcription factor [155,156]. The remaining component of AP-1, JUN, is phosphorylated by the JNK cascade of RAS signaling, which is also active in colon cancer [231]. Once formed, AP-1 leads to proliferation and survival by expressing genes such as *CCND1* and repressing *TP53* [232,233,234,235]. Therefore, WNT and RAS cooperate to express *CCND1*, by the concerted efforts of β-catenin/TCF4 and AP-1. It has been noted that WNT alone does not appear sufficient to fully express *CCND1* [236]. Additionally, RAS signaling, acting via ERK, leads to the phosphorylation of MYC and FOS, enhancing their transcriptional output [150,154]. Furthermore, ERK phosphorylates the SMAD proteins, which inhibits their nuclear accumulation and, therefore, their signaling capabilities [237,238]. Oncogenic RAS, therefore, aids WNT signaling in *CCND1* expression via AP-1 and MYC phosphorylation, helps to weaken TGF-β signaling by SMAD phosphorylation via ERK, and represses *TP53* via AP-1. The high levels of cyclin D, from WNT and RAS, bind to CDK4, a MYC target gene, allowing for the formation of cyclin D and CDK4 complexes [239]. CyclinD/CDK4 phosphorylates pRb, releasing the E2F factors [240,241]. MYC then functions alongside the E2F factors to facilitate binding at the *E2F* gene promoters, facilitating the activation of the E2F transcriptional program [242]. E2F target genes are necessary for DNA replication and the transition from G_1_ to S, such as *CCNE1* [243]. Cyclin E binds to CDK2, which further phosphorylates pRb, leading to the complete release of the E2F factors, and transition to the growth factor-independent stages of the cell cycle [214,244]. The concerted actions of WNT and RAS, therefore, generate a potent proliferative signal through the combined function of βcat/TCF4, MYC, and RAS/AP-1. Despite the combination of WNT and RAS, TGF-β appears to retain some proliferation inhibitory functions, as demonstrated by selection of TGF-β mutations. Its ability to repress *MYC* expression, as well as express *CDKN1A* and *CDKN2B*, may be the last checks on proliferation, which upon mutation of *SMAD4* or one of the TGF-β receptors, results in the complete release of *MYC* and unregulated proliferation of cancerous cells (Figure 4).

## 7. The Intestinal Microbiota

The microorganisms which reside in the colon are often overlooked in regard to their influence on colorectal cancer tumorigenesis. These organisms can generate a profound impact on the gut by the alteration of chemical species, interaction with intestinal cells, or by the release of toxins [245,246,247]. Given that intestinal polyps extrude into the lumen of the colon, an interaction between cells within the polyps and gut-resident microbes is expected. The pathogen, *C. difficile* secretes toxins which bind the FZD receptors, inhibiting their ability to bind a WNT ligand [248,249]. This inhibition of WNT signaling may result in extended wound-healing and inflammation. However, the actions of bacteria are not solely deleterious. Probiotic strains can compete against virulent bacterial species, protecting the intestines from inflammation and damage [250]. Evidently, much work is to be done to understand the capability of bacteria to influence intestinal health.

## 8. Conclusions

In colon cancer, the mutations of the WNT, RAS, and TGF-β signaling pathways recapitulate the signaling arrangement present in the intestinal stem cells. This stochastic recapitulation of a similar functional program indicates that the coordinated activities of these pathways results in a potent growth-inducing stimulus. These efforts are, at times, overlapping, such as the expression of *CCND1* through βcat/TCF4 and AP-1, whereas other efforts are complementary, such as *MYC* expression by WNT and MYC phosphorylation by RAS. TGF-β antagonizes the influences of WNT and RAS on the cell cycle by down-regulating *MYC* and up-regulating the cell cycle regulators *CDKN1A* and *CDKN2B*. The ability of TGF-β to regulate the cell cycle relies on the SMAD transcription factors, which are phosphorylated by the activated RAS pathway to inhibit their nuclear translocation. As colon cancer develops, mutations in the WNT pathway lead to an increase of MYC, which results in small adenoma formation. The coupling of oncogenic RAS to deregulated WNT leads to the formation of a large adenoma, as βcat/TCF4 and MYC are complemented by AP-1 of RAS. Loss of TGF-β, the developmentally designated gatekeeper, allows for the unrestrictive action of MYC and RAS. Taken together, the WNT, RAS, and TGF-β mutations function cooperatively to form a malignant cell.
